# How Much Is Too Much? The Influence of Work Hours on Social Development: An Empirical Analysis for OECD Countries

**DOI:** 10.3390/ijerph16244914

**Published:** 2019-12-05

**Authors:** Bei Liu, Hong Chen, Xin Gan

**Affiliations:** School of Management, China University of Mining and Technology, Xuzhou 221116, China; liubeii@163.com (B.L.); aifusengan@126.com (X.G.)

**Keywords:** work hours, individual-organizational-social perspective, health quality, organizational performance, economic development

## Abstract

Work is a cornerstone of social development. Quantifying the impact on development of fluctuations in work hours is important because longer work hours increasingly seem to be the norm. Based on an integrative perspective that combines individual, organizational, and social factors, we constructed a model using data from 31 member countries of the Organisation for Economic Co-operation and Development (OECD). The proposed model was used to test the effect of work hours on different levels and to propose feasible suggestions accordingly. The results show that people in developing countries work more hours per week than those in developed countries, and that males work longer hours than females. Furthermore, regression analysis shows that current work hours are having a negative impact on development in OECD countries, especially in developing countries where people are working longer hours. Longer hours, in other words, do not promote development effectively. Specifically, work hours at the individual level are negatively related to health. At the level of organization, work hours are a reverse indicator of organizational performance, and at the level of society, there is a negative relationship between work hours and economic development. This study provides support for the proposition by the International Labour Organization to reduce work hours, and it facilitates our understanding of the relationship between work hours and social development.

## 1. Introduction

There is a popular belief that working long hours promotes social development. During the industrial revolution, organisations exploited cheap labour and hired employees as “working machines, putting them to work in countless sweatshops” [1]. Even now, emphasis is often placed more on work hours than the tasks themselves, whereby work hours serve as a common indicator of employee performance [2]. The world has gradually moved toward a so-called 24-h society [3], and some cultures (e.g., Japan) endorse long work hours, regarding it as normal for employees to work overtime [4]. However, there is evidence that extreme work hours negatively affect health and organizational performance [5], and questions have been raised about the trade-off between longer work hours and increased income on the one hand [6,7], and well-being and physical health on the other [8,9,10]. However, most previous studies focused on the effect of work hours on the organization and the individual. Few have sought to understand this issue from a more extensive and comprehensive perspective [11].

Social development pertains to economic and social changes in a broad sense, e.g., through the establishment of institutions and economic and political planning [12]. Social development theory provides a framework to understand qualitative changes in society while offering ways to realize a better world [13]. Several questions remain, however: Do current work hours perfectly match the pace of social development? What is the real impact of changes in work hours on social development? How can the real relationship between work hours and social development be analysed comprehensively? To answer these questions, is important to clarify the relationship between work hours and social development. The perspective of individual-organizational-social may be helpful [14,15]. Liu et al. adopted such a perspective to analyse the evolution of work hours, providing a comprehensive, hierarchical and systematic framework for understanding the effect of work hours [11,16]. By integrating individual, organizational and social factors, studies on work hours from an individual perspective mostly focus on the interaction between a hours spent at work and some physical or psychological variable over a specified period, such as the relationship between overtime and mental health [17]. Studies on work hours from an organizational perspective tend to concentrate on the development of the organizations themselves, such as the relationship among overtime, firm productivity and innovation [18]. The study of work hours from a social perspective aims to analyse the economic and social environment of work hours, and to locate the effect of work hours on social development [19]. Therefore, the study of work hours from these three perspectives is not limited to specific individual or organizational variables. Rather, an integrative approach is hierarchical, extensive and comprehensive.

At the individual level, the relationship between work and health is a core issue for workplace management [20]. Long work hours can erode the health of employees and cause physical illnesses, such as cerebrovascular diseases [21,22]. These irreversible physiological impairments accelerate the depletion of an employee’s resources, decrease quality of life and burden society with medical expenditures. In light of this, we proposed the following hypothesis:

**Hypothesis 1** **(H1).** *Work hours are significantly associated with individual health.*

At the organizational level, the performance of the organization is a crucial matter and is largely dependent on employee work [23,24]. In industry especially, manual operations performed by workers are the decisive factor for organizational performance [25], which means that it is necessary to guarantee the work hours of employees to maintain production. However, previous research has suggested that the direct physiological damage caused by long work hours indirectly decreases organizational performance [26]. Furthermore, employees tend to be absent or quit when they cannot afford to work longer hours [27], once again negatively influencing organizational performance. With industrial automation and information technology gradually replacing the original manual manufacturing paradigm, it is worth asking whether there has been any change in the absolute dependence of organizations on individual work hours. To clarify the real relationship between work hours and organizational performance, we proposed a second hypothesis:

**Hypothesis 2** **(H2).** *Work hours are significantly associated with organizational performance.*

At the level of society, economic development is regarded as an important indicator of social development [28]. Previous studies on work hours mostly consider labour to play an important role in economic development [29]. With the rapid development of information technology, however, it is pertinent to determine whether the current number of hours employees work promotes economic growth, especially given the negative effects of long work hours. Therefore, we proposed a third hypothesis to explore the true relationship between these two factors:

**Hypothesis 3** **(H3).** *Work hours are significantly associated with economic development.*

According to an integrative perspective that combines individual, organizational and social factors, the relationships among these three levels are hierarchical and comprehensive [15]. Thus, we propose a final hypothesis:

**Hypothesis 4** **(H4).** *The relationships among the individual, organization, and society are hierarchical, proceeding from the individual to society.*

**Figure 1:**
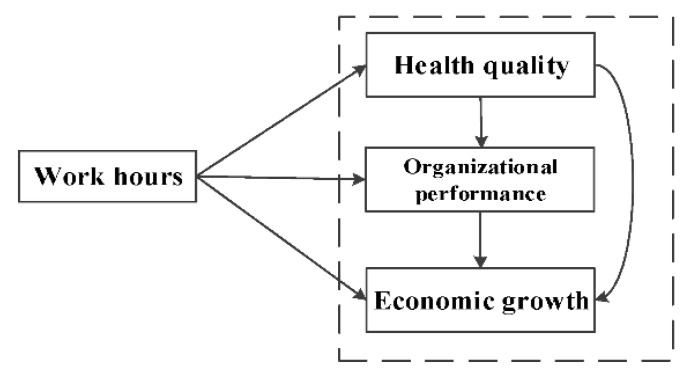
Theoretical model of this study.

As shown in Figure 1, the main objectives of this study were to compare the relationship between work hours and development in different countries based on an integrated perspective, and to quantify the relationship between existing work hours and development from the perspective of the individual, organization and society. Our study highlights the relationship between work hours and development, and we expect that our results will provide theoretical support to organizations and nations when devising policy around work hours.

## 2. Materials and Methods

### 2.1. Model Construction

A fixed-effect regression model was used to analyse the mechanisms of the interaction of work hours with individual health quality, organizational performance and economic development. Specifically, we used a three-stage least-square (3SLS) estimation of instrumental variables to explore the relationship among them, and the growth rate of variables at lag two was chosen as a tool variable for each variable at lag one. In terms of individual health, the concept of life expectancy was first proposed by Sanders, who referred to it as a new health indicator that combined mortality and morbidity [30]. It has become a commonly used measure of individual health [31]. Thus, in this study, the life expectancy of individuals (collected from data issued by the World Bank [32]) was selected as the measurement index of individual health. Control variables such as the youth dependency ratio were also added to the model. The specific model expression as follows:

(1) $$H{\mathrm{ealth}}_{\mathrm{it}}=\alpha_{0}+\alpha_{1}\ln GDP_{it}+\alpha_{2}\ln Time_{it}+\alpha_{3}\ln H_{it}+\alpha_{4}\ln Young_{it}+\alpha_{5}\ln Old_{it}+\alpha_{6}\ln S_{it}+\eta_{it}$$

This equation was used to test Hypothesis 1. In Equation (1), the real per capita gross domestic product (*GDP*) of countries in 2010 (in constant U.S. dollars) was used to indicate the quality of national economic development. In Equation (1), *Time_it_* denotes average work hours in country *i* in year *t*, *Young_it_* represents the juvenile dependency ratio of that country in that year, *Old_it_* represents the old-age dependency ratio of that country in that year, *S_it_* represents the savings rate of that country in that year, and *α*_0_ and *η_it_* denote the fixed effect and disturbance of the corresponding equation, respectively, where *α*_1_–*α*_6_ are the estimated coefficients of the corresponding equation.

In terms of organizational performance, the total factor productivity (TFP) refers to the main output of each factor of the production unit [33], which is used as a common indicator to measure organizational performance [34]. Hence, *TFP* was chosen as the indicator of organizational performance to explore the impact of work hours. The specific model to test Hypothesis 2 is as follows:

(2) $$TFP_{it}=\beta_{0}+\beta_{1}GDP_{it}+\beta_{2}Time_{it}+\beta_{3}H_{it}+\beta_{4}E_{it}+\beta_{5}I_{it}+\beta_{6}Infra_{it}+\mu_{it}$$

In Equation (2), the set of country-specific variables was controlled to minimize errors from omitted variables. These variables include financial development (*E*, the proportion of total stock transactions to GDP), openness (*F*, the proportion of net foreign direct investment to GDP), human capital (*H,* the enrolment rate in colleges and universities), industrial development (*I*, the proportion of industrial value added to GDP) and infrastructure (*Infra*, per-capita electricity consumption). Moreover, *β*_0_ and *μ_it_* represent the fixed effect and disturbance of the corresponding equation, respectively, and *β*_1_–*β*_6_ represent the estimated coefficients of the corresponding equation.

At the social level, economic development is influenced by various factors, including individual development and organizational performance [35]. Therefore, we incorporated Equation (1), which measures individual health quality, and Equation (2), which measures organizational performance, into an economic development model to explore the impact of work hours, individual health and organizational performance on economic development. The specific model to test Hypotheses 3 and 4 is as follows:

(3) $$GDP_{it}=\gamma_{0}+\gamma_{1}\ln Time_{it}+\gamma_{2}\ln H_{it}+\gamma_{3}\ln E_{it}+\gamma_{4}\ln I_{it}+\gamma_{5}\ln FDI_{it}+\gamma_{6}\ln RD_{it}+\gamma_{7}\ln Infra_{it}+\varepsilon_{it}$$

In Equation (3), *RD* indicates government research and development investment as a proportion of GDP, and *γ*_0_ and *ε_it_* denote the fixed effect and the perturbation term of the equation. Respectively, *γ*_1_–*γ*_7_ are the estimated coefficients of the equation.

### 2.2. Data Resources

The observed growth in the world economy suggests fierce international competition, reinforcing the connections among countries. The development status of Organisation for Economic Co-operation and Development (OECD) countries effectively reflects the relationship between work hours and social development because its member states are at different levels of development. We selected data regarding work hours, economic development, etc. from 31 typical OECD countries as research samples to ensure consistency and comparability. Other development indices, such as GDP, TFP and life expectancy, were taken from the World Bank database [32]. Table 1 shows the explanatory variables, core explained variables and control variables.

## 3. Results

### 3.1. Descriptive Statistics for Work Hours in OECD countries

**Figure 2:**
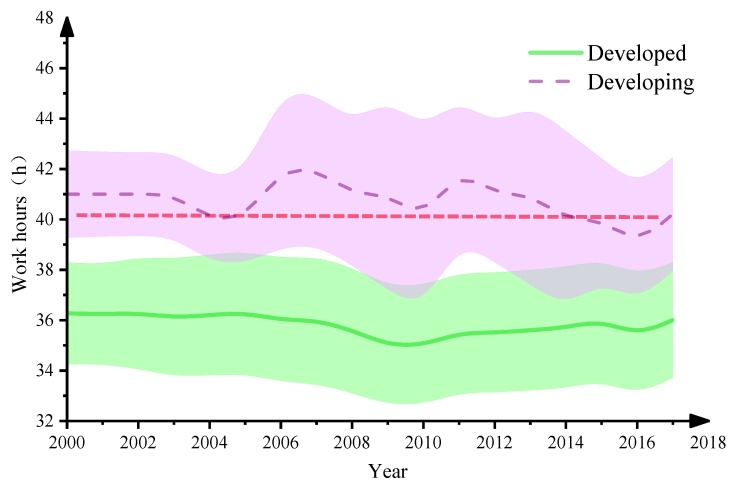
Weekly hours worked in different OECD countries.

The 2018 United Nations publication *Human Development Indices and Indicators* classifies countries as developing or developed according to comprehensive indicators such as life expectation, education level and economic development level [36]. According to the catalogue of developing regions provided by this publication [36], the descriptive analysis of 31 OECD countries can be divided into developed countries (such as the United States, Britain, Sweden and Japan) and developing countries (such as Mexico, Latvia and Turkey). Figure 2 shows the mean hours worked per week in different countries.

Figure 2 shows a decreasing tendency in the mean hours worked per week in OECD countries. The average number of work hours per capita in OECD developing countries is higher than in developed countries. In addition, the shaded part in Figure 1 denotes the standard deviation of work hours in countries with different levels of development, reflecting considerable fluctuation and less stability in developing countries than in developed countries.

Furthermore, we compared the weekly work hours (work hours per person per week) in some developed and developing countries to clarify further the differences in work hours among countries. The results indicate that developing countries have higher average weekly work hours than developed countries. According to the modest work standard of the International Labour Organization (ILO), an employee should work no more than 40 h per week. Our results show that OECD developing countries exceed this number on average, whereas OECD developed countries fall below it on average.

**Figure 3:**
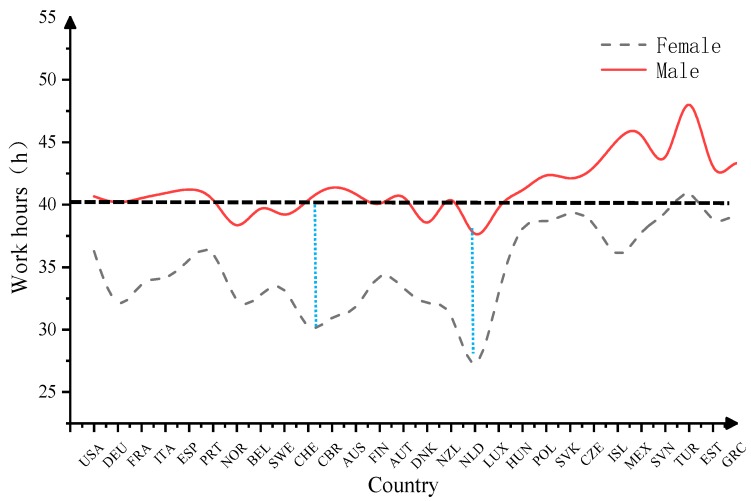
Weekly work hours of men and women in OECD countries. Note: USA-the United States of America; DEU-Germany; FRA-France; ITA-Italy; ESP-Spain; PRT-Portugal; NOR-Norway; BEL-Belgium; SWE-Sweden; CHE-Switzerland; AUS-Australia; FIN-Finland; AUT-Austria; DNK-Denmark; NZL-New Zealand; NLD-Netherlands; LUX-Luxembourg; HUN-Hungary; POL-Poland; SVK-Slovak Republic; CZE-Czech Republic; ISL-Iceland; MEX-Mexico; SVN-Slovenia; TUR-Turkey; EST-Estonia; GRC-Greece.

The gender differences in weekly work hours in OECD countries were also compared. As shown in Figure 3, men in OECD countries worked more hours each week than women. The blue line in Figure 3 denotes a 10-h difference to measure the gender differences in work hours in various countries. The results show that the gender differences in work hours are conspicuous in the Netherlands and Switzerland, with differences of more than 10 h, whereas there are less gender differences in Hungary, Slovakia and Latvia. Furthermore, according to the ILO’s standard of no more than 40 work hours per week, men in OECD countries worked more overtime, especially in Mexico, Turkey and the United Kingdom.

### 3.2. Work Hours and Individual Health

As explained above, individual health is represented by individual life expectancy in our study. In Equation (1), work hours and life expectancy are explanatory variables. These variables were incorporated into the model to explore the dynamic relationship between them. In addition, the effects of control variables such as gender and the level of economic development were also considered. The regression coefficients of the effect of work hours on the utility of independent variables and their significance are provided in each table.

Six models were constructed to test Hypothesis 1, as shown in Table 2: A model of total work hours and life expectancy (Column 1), and models of work hours and life expectancy of different gender groups (Column 2), and of different development types (Columns 3 and 4). Furthermore, because life expectancy affects the national GDP, we also tested the relationship between life expectancy and GDP (Column 5). The relationship between work hours and individual life expectancy in OECD countries is reported in Column 1 and suggests a negative relationship between work hours and life expectancy at the 5% level (−0.0072, *p* < 0.001). Hence, we expect that decreasing work hours in OECD countries will improve health quality, verifying Hypothesis 1.

Column 2 in Table 2 reports the association between work hours and life expectancy from the perspective of gender, after weighing the proportion of male and female workers. The results show that the effect of work hours on the health quality of men (−0.0057, *p* < 0.001) is stronger than on women (−0.0012, *p* < 0.00). In terms of different levels of development, Column 3 and Column 4 indicate that the association between weekly work hours and health in developing countries (−0.0107, *p* < 0.001) is stronger than in developed countries (−0.0033, *p* < 0.001). In addition, Column 5 suggests a significant positive relationship between individual health quality and economic development (*p* < 0.001). The test results with one lag were similar to those with one lag, indicating a stable relationship.

### 3.3. Work Hours and Organizational Performance

The TFP was adopted as an indicator of organizational performance, according to Equation (2), to explore the impact of work hours on organizational development, taking into account gender effects and status [37].

Six models were constructed to test Hypothesis 2, as shown in Table 3: A model of total work hours and TFP (Column 1), and models of work hours and TFP of different gender groups (Column 2) and different development types (Columns 3 and 4). Furthermore, because TFP affects the national GDP, we also tested the relationship between TFP and GDP (Column 5). Table 3 shows the results for Hypothesis 2 using panel data from OECD countries from 2000 to 2016. In the table, Column 1 reports that work hours (an explanatory variable) have a negative impact on organizational performance (−0.0153, *p* < 0.001). Therefore, Hypothesis 2 is verified. Column 2 further reports this impact by gender difference. The results show that more work hours by females can promote organizational performance (0.0005, *p* < 0.001), and that a decrease in male work hours is more conducive to organizational performance (−0.0005, *p* < 0.001).

Columns 3 and 4 both indicate that work hours in developed countries promote organizational performance (0.0006, *p* < 0.001). However, current work hours in developing countries are detrimental to organizational performance (−0.0002, *p* < 0.001). The regression coefficients show that control variables such as financial development, human capital, and industrial development have a significant relationship with organizational performance (*p* < 0.001) and that there is a positive correlation between organizational performance and economic development (*p* < 0.001). The test results with one lag were similar to those with no lag, indicating a stable relationship.

### 3.4. Work Hours and Social Development

From a social viewpoint, GDP per capita is an effective tool to understand the macroeconomic operation of a country or region, and it is often used as an indicator of economic development [38]. Therefore, it was incorporated as a dependent variable into the equation model to explore the impact of work hours. At the same time, the impact of gender, status and other control variables on this relationship was considered.

Six models were constructed to test Hypothesis 3, as shown in Table 4: A model of total work hours and GDP (Column 1), and models of work hours and GDP of different gender groups (Column 2) and different development types (Columns 3–5). According to Table 4, work hours in OECD countries had a negative relationship with economic development (−0.0019, *p* < 0.001), which means that a unit increase in work hours may not achieve a significant increase in economic development. This verifies Hypothesis 3. In addition, the work hours of men had a significantly negative relationship with economic development (−0.0022, *p* < 0.001), although the relationship between economic development and the number of hours worked by women had a significantly positive correlation (0.0009, *p* < 0.001). Furthermore, the test results with one lag were similar to those with no lag, indicating a stable relationship.

**Figure 4:**
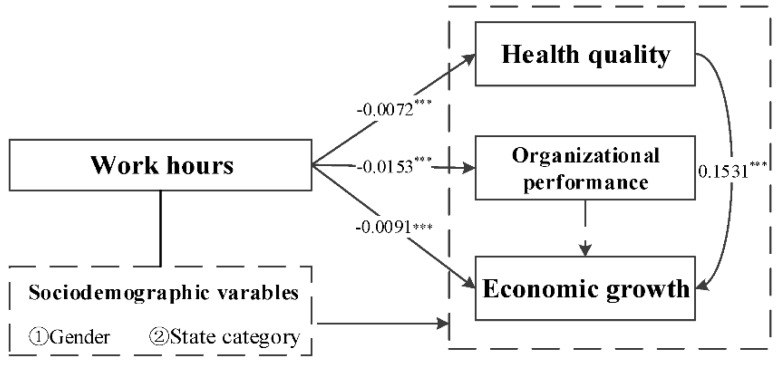
Empirical results of the hierarchical impact of work hours. Note: *** 1% significance level; ** 5% significance level; * 10% significance level.

Combining the results of this study, it can be concluded that work hours have a negative relationship with the development of individual, organizational, and social variables. As such, Hypothesis 4 is also verified. Specifically, current work hours cannot effectively promote the quality of individual health, and longer working hours merely play a weak role promoting organizational performance, let alone maintaining effective economic growth (Figure 4). At the same time, the results show that individual levels of health in OECD countries are positive predictors of economic development, and that the impact of organizational performance on economic development is not significant.

## 4. Discussion

### 4.1. Impact of Work Hours

The findings of this study confirm that current work hours in OECD countries are not conducive to individual health. In addition, as the basic unit of social development, the quality of individual development is directly related to the overall development of society [39]. In general, long work hours are not conducive to individual physical and mental health [40]. However, previous studies on the relationship between work hours and physical health were inconsistent. Although some studies have shown that work hours function as a risk indicator to employees [22], other studies suggested that the relationship between work hours and physical health is not significant. We believe that the reason for these different conclusions pertains to the generalisability of the samples. In our study, we selected official data on work hours from 31 OECD countries over the past 20 years. The data we used is thus highly representative, objective and authoritative, and this should render the results more convincing.

At the organizational level, there is a negative correlation between work hours and organizational performance. In other words, relying solely on increases in individual work hours will not promote organizational performance. Andreja et al. also revealed a causal link between work hours and economic development in European Union countries [41]. As Glosser and Golden suggested, the relationship between work hours and industrial employment levels, as well as industry output, had already begun to weaken by 1979 [29]. A possible explanation for these results is that the current number of hours worked has reached excessive levels in terms of the actual demands for improving efficiency. Previous studies have shown that long work hours contribute to burnout and even suicide [42,43,44]. Indeed, employee loss would decrease organizational performance [45]. This study provides powerful support for relevant policies seeking to constrain work hours in Italy and Finland [46,47].

At the social level, the results of regression analysis show an inverse relationship between work hours and economic development, indicating that an appropriate reduction in work hours is more conducive to socioeconomic development. Thus, guiding the work values of the upper social strata and establishing a demonstrative model is more effective than reducing individual work hours, because, as Cowling argues, social comparison, contagion and structure are the main causes of a culture of long work hours [48]. In addition, we found that an increase in female work hours is more conducive to economic growth than an increase in male work hours. Previously, a survey conducted by the World Health Organization showed that there are significant gender differences in work hours, and that the work hours and wages of males are much higher than those of females [49]. Bøler et al. also indicated that employers tend to recruit males for some particular positions because their work hours are more flexible than those of females [50]. It seems that males are more profitable for the development of an organization than females. However, our results show that increasing female work hours can promote economic development. Thus, mitigating gender differences in terms of work hours is an urgent matter. Indeed, work hours are considered an important indicator of national economic development and labour development [51]. However, our results show a marginally diminishing effect of work hours on economic growth: If the input factor of work hours increases continuously and equally to a certain output, the production efficiency will decline [52]. Therefore, reducing work hours appropriately will not hinder the rate of economic growth.

Furthermore, compared to the work hours of women, the work hours of men achieve a higher per capita baseline, and their contribution to economic benefits is more marginally diminished than that of women. The leisure economy may explain this phenomenon. The leisure economy, as a replacement for the work-hour economy, plays an important role in economic development [53]. It is believed that the absolutely dominant position of work in individual life will weaken in the future, and that a reasonable combination of work and leisure hours will be more conducive to long-term economic development.

### 4.2. Analysis of Differences between Work Hours and Levels of Development among Countries

The findings of this study demonstrate that the average work hours per capita in OECD developing countries are higher than those of developed countries, and that longer work hours in developing countries predict their development inversely from the perspective of individual, organizational and social factors. For developed countries, current work hours have a positive impact on organizational performance and economic development. We believe that this situation occurs mainly because of differences in industrialization and technological innovation between these two groups of countries. Developing countries in the OECD take advantage of their labour force by developing labour-intensive industries to reduce production costs from low levels of industrialization and technological innovation [54]. The result is a rigid requirement for more individual work hours.

By comparison, developed countries have higher levels of technological innovation, with higher per capita output per hour [55]. Therefore, they work relatively fewer hours. Furthermore, developing countries have several problems in terms of protecting labour rights, poor working conditions [56] and labour market segmentation [57]. These factors put pressure on individual worker to work longer hours. With reference to the findings of this study, we propose that the positive relationship between work hours and social development is becoming saturated, especially in developing countries. Consequently, priority should be placed on improving the level of technological innovation rather than extending work hours. Moreover, reducing work hours appropriately can be more beneficial to development. It would be beneficial for developing countries to provide reasonable policy guidance for reducing work hours, because several countries have promulgated laws to restrict organizational behaviour to protect employee rights with regard to work hours, such as the 405 Regulations issued by New York State to restrict resident work hours and labour legislation reform in Finland [47,58].

We found that men in OECD countries worked longer hours than women and that the proportion of men working overtime was higher. Generally, the traditional role of women is to care for the family [59]. Yet, the work hours of women have been increasing steadily due to more female workers entering the workplace, combined with economic development and greater public awareness [60]. For these reasons, women have suffered more conflict between work and family than males [61]. In this context, large numbers of women opt to reduce their work hours in order to balance work and family life [62]. On the whole, the average work hours of women were thus lower than those of males, and the proportion of overtime work by women was also lower.

Some limitations should be noted when considering this study. We exclusively analysed the impact and utility of work hours with regard to OECD countries. The impact of work hours on non-OECD countries remains to be determined. Further, we adopted a comprehensive and typical perspective of individual, organizational, and social factors to analyse work hours, although additional research perspectives should be considered as well. Finally, the indicators of the impact and utility of work hours need to be explored further. For example, cultural differences and the various political structures of countries should be considered.

## 5. Conclusions

This study explored the relationship among work hours, individual health, organizational performance, and economic development. Our results show that current work hours at the individual level are negatively associated with health, and that this negative relationship between work hours and health is stronger in men than in women. At the organizational level, work hours are negatively associated with organizational performance. At the social level, there is a negative relationship between work hours and economic development. These findings suggest that current work hours cannot effectively promote the overall progress of society, and that a decrease in work hours would be more conducive to improvements in this area.

## Figures and Tables

**Table 1:** Statistical description of variables.

Variable	Mark	Unit	Date Resource	N	Mean	SD
Per capita GDP	*GDP*	ten thousand	World Bank Database (date to April 2018)	527	3.83	2.21
Work hours	*WH*	H/year	OECD Database (date to April 2018)	527	1734.96	197.78
Total factor productivity	*TFP*	--	Federal Reserve Economic Database (date to April 2018)	527	0.88	0.21
Life expectancy	*LE*	--	World Bank Database (date to April 2018)	527	78.81	3.03
Financial development	*E*	%	World Bank Database (date to April 2018)	527	50.10	54.85
Openness	*F*	%	World Bank Database (date to April 2018)	527	5.54	14.66
Human capital	*H*	%	World Bank Database (date to April 2018)	527	63.90	18.13
Industrial development	*I*	%	World Bank Database (date to April 2018)	527	0.24	0.05
Government research and development	*RD*	%	World Bank Database (date to April 2018)	527	1.76	0.88
Infrastructure	*Infra*	Tens of millions of hours	World Bank Database (date to April 2018)	527	0.93	0.80
Juvenile dependency ratio	*Young*	%	World Bank Database (date to April 2018)	527	17.56	3.82
Old-age dependency ratio	*Old*	%	World Bank Database (date to April 2018)	527	23.36	5.53
Savings rate	*S*	%	World Bank Database (date to April 2018)	527	23.29	6.15

**Table 2:** Results of work hours and individual health quality.

	(1)	(2)	(3)	(4)	(5)	(6)
	Time→Life				Life→GDP	
	Whole	Whole	Developed	Developing		
	Life	Life	Life	Life	GDP	GDP
*WH*	−0.0072 *** (−6.14)		−0.0033 ** (−2.02)	−0.0107 *** (−6.53)		
*M-WH*		−0.0057 *** (−5.75)				
*F*-*WH*		−0.0012 *** (−1.41)				
*GDP*	1.2017 *** (7.81)	0.9164 *** (6.55)	1.3836 *** (7.92)	1.0712 *** (3.81)		
*H*	0.0219 *** (5.36)	0.0198 *** (4.17)	0.0309 *** (5.53)	0.0123 ** (2.23)		
*Young*	−0.2242 *** (−4.88)	−0.2601 *** (−4.96)	−0.3649 *** (−4.43)	−0.1566 *** (−3.00)		
*Old*	0.2254 *** (13.59)	0.2683 *** (13.93)	0.1226 *** (7.22)	0.4497 *** (14.73)		
*S*	−0.0195 * (−1.71)	−0.0395 *** (−3.07)	−0.0895 *** (−6.63)	0.0306 (1.50)		
*EL*					0.1531 *** (14.68)	
*L.-Life*						0.1516 *** (14.90)
*Cons*	84.4830 *** (34.80)	80.4571 *** (29.10)	82.0118 *** (26.97)	86.4199 *** (23.44)	−9.6120 *** (−7.67)	−10.7864 (−8.72)
*Control*	Yes	Yes	Yes	Yes	Yes	Yes
*Country effect*	Yes	Yes	Yes	Yes	Yes	Yes
*Tine effect*	Yes	Yes	Yes	Yes	Yes	Yes
*N*	527	527	346	181	527	496
*R* ^2^	0.77	0.76	0.82	0.88	0.50	0.48

Note: *GDP* represents the gross domestic product; *WH* represents weekly work hours; *M-WH* represents weekly work hours of men; *F-WH* represents weekly work hours of women; *TFP* represents the proportion of net foreign direct investment to *GDP*; *LE* represents life expectancy; *E* represents the proportion of total stock transactions to GDP; *F* represents the level of openness; *H* represents human capital; *I* represents industrial development; *Infra* represents infrastructure; *Time_it_* represents average work hours in country *i* in year *t*; *Young_it_* represents the juvenile dependency ratio of that country in that year; *Old_it_* represents the old-age dependency ratio of that country in that year; and *S_it_* represents the savings rate of that country in that year. *** 1% significance level; ** 5% significance level; * 10% significance level.

**Table 3:** Results of work hours and organizational performance.

	(1)	(2)	(3)	(4)	(5)	(6)
	Whole	Whole	Developed	Developing	TFP→GDP	
	TFP	TFP	TFP	TFP	GDP	GDP
*WH*	−0.0153 *** (−3.99)		0.0006 *** (4.38)	−0.0002 * (−1.69)		
*M-WH*		0.0005 *** (5.35)				
*F-WH*		−0.0005 *** (−3.37)				
*GDP*	0.0210 * (1.78)	0.0305 *** (2.66)	0.0119 (0.75)	0.0911 *** (4.27)		
*H*	0.0001 (0.40)	0.0012 *** (3.16)	0.0005 (0.81)	−0.0013 *** (−5.05)		
*E*	0.0002 * (1.87)	0.0001 (1.16)	0.0002 * (1.66)	−0.0003 (−1.13)		
*I*	0.7978 *** (6.56)	0.4779 *** (3.74)	0.4347 *** (2.79)	1.0503 *** (6.38)		
*Infra*	0.0391 *** (3.31)	0.0308 *** (2.77)	0.4104 *** (5.40)	0.2421 *** (4.27)		
*TFP*					0.1133 (0.64)	
*L.TFP*						0.2396 (1.39)
*Cons*	−0.0744 (−0.39)	0.3389 * (1.67)	−0.6864 ** (−2.38)	0.5411 *** (2.74)	2.6626 *** (13.93)	2.5983 *** (13.63)
*Control*	Yes	Yes	Yes	Yes	Yes	Yes
*Country effect*	Yes	Yes	Yes	Yes	Yes	Yes
*Tine effect*	Yes	Yes	Yes	Yes	Yes	Yes
*N*	527	527	346	181	527	496
*R* ^2^	0.15	0.21	0.23	0.53	0.24	0.20

Note: GDP represents the gross domestic product; *WH* represents weekly work hours; *M-WH* represents weekly work hours of men; *F-WH* represents weekly work hours of women; *TFP* represents the proportion of net foreign direct investment to GDP; *LE* represents life expectancy; *E* represents the proportion of total stock transactions to GDP; *F* represents the level of openness; *H* represents human capital; *I* represents industrial development; *Infra* represents infrastructure; *Time_it_* represents the average work hours in country *i* in year *t*; *Young_it_* represents the juvenile dependency ratio of that country in that year; *Old_it_* represents the old-age dependency ratio of that country in that year; *S_it_* represents the savings rate of that country in that year. *** 1% significance level; ** 5% significance level; * 10% significance level.

**Table 4:** Results of work hours and economic development.

	(1)	(2)	(3)	(4)	(5)	(6)
	Whole	Whole	Developed		Developing	
	GDP	GDP	GDP	GDP	GDP	GDP
*WH*	−0.0019 *** (−4.93)		−0.0027 *** (−5.19)		0.001 ** (2.52)	
*M-WH*		−0.0020 *** (−5.48)		0.0060 *** (6.86)		−0.0007 *** (−2.68)
*W-WH*		0.0002 *** (0.38)		−0.0026 *** (−5.52)		−0.0022 *** (−5.27)
*RD*	0.0596 (1.19)	0.1001 ** (2.05)	−0.131 * (−1.74)	0.0223 (0.33)	0.125 *** (3.16)	0.0490 * (1.34)
*H*	0.0078 *** (5.88)	0.0049 *** (3.18)	0.014 *** (5.75)	0.006 *** (2.53)	0.002 * (1.76)	0.0017 * (1.61)
*E*	0.0006 (1.59)	0.0007 ** (1.98)	0.001 * (1.85)	0.0014 *** (3.75)	0.002 * (1.70)	0.0017 * (1.64)
*FDI*	−0.0003 (−0.45)	−0.0002 (−0.24)	−0.001 (−1.22)	−0.0010 (−0.38)	−0.001 (−0.49)	0.000 (0.28)
*I*	0.4224 (0.90)	1.2536 ** (2.49)	−0.690 (−1.20)	1.2082 *** (2.16)	3.989 *** (7.92)	3.806 *** (7.08)
*Infra*	0.0528 (1.08)	0.1001 ** (2.18)	−0.090 (−1.62)	0.0185 (0.39)	3.617 *** (12.27)	3.074 *** (10.69)
*Cons*	6.3374 *** (8.48)	5.1155 *** (7.96)	9.065 *** (9.02)	2.576 *** (2.41)	−2.908 *** (−4.08)	2.032 *** (2.98)
*Control*	Yes	Yes	Yes	Yes	Yes	Yes
*Country effect*	Yes	Yes	Yes	Yes	Yes	Yes
*Tine effect*	Yes	Yes	Yes	Yes	Yes	Yes
*R* ^2^	0.28	0.29	0.28	0.26	0.24	0.36

Note: *GDP* represents gross domestic product; *WH* represents weekly work hours; *M-WH* represents weekly work hours of men; *F*-*WH* represents weekly work hours of women; *TFP* represents the proportion of net foreign direct investment to *GDP*; *LE* represents life expectancy; *E* represents the proportion of total stock transactions to GDP; *F* represents the level of openness; *H* represents human capital; *I* represents industrial development; *Infra* represents infrastructure; *Time_it_* represents the average work hours in country *i* in year *t*; *Young_it_* represents the juvenile dependency ratio of that country in that year; *Old_it_* represents the old-age dependency ratio of that country in that year; and *Sit* represents the savings rate of that country in that year. *** 1% significance level; ** 5% significance level; * 10% significance level.
